# Transient left ventricular apical ballooning and exercise induced hypertension during treadmill exercise testing: is there a common hypersympathetic mechanism?

**DOI:** 10.1186/1476-7120-6-37

**Published:** 2008-07-18

**Authors:** Abhijeet Dhoble, Sahar S Abdelmoneim, Mathieu Bernier, Jae K Oh, Sharon L Mulvagh

**Affiliations:** 1 Echocardiographic Laboratory, Division of Cardiovascular Medicine, Mayo Clinic, Rochester, Minnesota, USA; 2 Division of Internal Medicine, Michigan State University, East Lansing, Michigan, USA

## Abstract

**Objective:**

To describe two cases of Takotsubo like myocardial contractile pattern during exercise stress test secondary to hypertensive response.

**Background:**

Treadmill exercise testing is known to cause sympathetic stimulation, leading to increased levels of catecholamine, resulting in alteration in vascular tone. Hypertensive response during exercise testing can cause abnormal consequences, resulting in false positive results.

**Cases:**

We present the cases of two patients experiencing apical and basal akinesis during exercise stress echocardiography, in whom normal wall motion response was observed on subsequent pharmacologic stress testing. The first patient developed transient left ventricular (LV) apical akinesis during exercise stress echocardiography. Due to high suspicion that this abnormality might be secondary to hypertensive response, pharmacologic stress testing was performed after three days, which was completely normal and showed no such wall motion abnormality. Qualitative assessment of myocardial perfusion using contrast was also performed, which showed good myocardial blood flow, indicating low probability for significant obstructive coronary artery disease. The second patient developed LV basal akinesis as a result of hypertensive response during exercise testing. Coronary angiogram was not performed in either patient due to low suspicion for coronary artery disease, and subsequently negative stress studies.

**Results:**

Transient stress induced cardiomyopathy can develop secondary to hypertensive response during exercise stress testing.

**Conclusion:**

These cases provide supporting evidence to the hyper-sympathetic theory of left ventricular ballooning syndrome.

## Background

Treadmill exercise testing is known to cause a hypersympathetic state, leading to catecholamine excess, resulting in increased heart rate (HR) and blood pressure (BP) [1]. False positive results with exercise stress testing have been attributed to hypertensive response during stress [1,2]. Although the pathogenesis is unclear, possible mechanisms include subendocardial ischemia due to excessive intraventricular pressures, and associated dynamic left ventricular (LV) outflow tract obstruction leading to reduced cardiac output and coronary perfusion. Sudden catecholamine surges leading to abnormal ventricular contraction may also contribute to wall motion abnormalities (WMA) [2]. A similar mechanism has been proposed for the pathogenesis of stress induced cardiomyopathy, also known as Takotsubo cardiomyopathy (TC), or transient LV apical ballooning [3,4]. Recently, there has also been case reports of 'inverted Takotsubo' syndrome where the base and mid portion of LV reveal WMA as opposed to apical segments [5].

We present two cases in this report: one with transient LV apical akinesis and second with LV basal akinesis during treadmill exercise testing.

## Case 1

**Figure 1:**
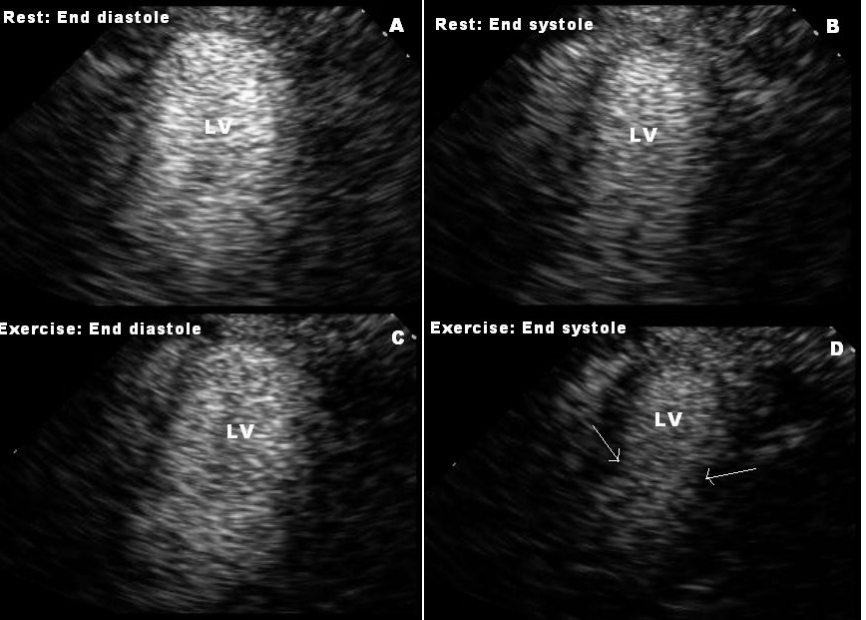
**Apical 2-chamber views taken during treadmill exercise testing, demonstrating mid to apical akinesis which develops during stress**. Images A and B are taken during rest, and C and D are at peak stress, during diastole (Images A and C) and systole (Images B and D) Image D shows mid to apical akinesis, arrows indicate "hinge point" between basal hyperkinesis and mid to apical akinesis. (LV: Left ventricle).

A 50 year old woman with dyslipidemia underwent exercise echocardiography testing with standard Bruce protocol to evaluate her chest pain. She described her chest pain as dull, aching, central 5/10 chest pain, lasting for about a minute when she was lying down after her dinner three days prior to test. This pain never recurred after that one isolated incidence. Patient's only medication was atorvastatin 10 mg daily. During exercise stress test, she achieved 7.2 metabolic equivalents (METS) during the protocol. New WMA were noted in the mid and apical segments, with apical segments being akinetic and mid segments being hypokinetic (figure 1). Please refer to table 1 for the detailed result of stress echocardiogram. The abnormal stress result was suspected to be secondary to hypertensive response, and pharmacologic stress testing with myocardial perfusion study was requested for further clarification. Dobutamine stress echocardiography (DSE) was performed three days later with contrast infusion according to standard protocol. The study was completely normal, and failed to demonstrate any abnormality in wall motion. Cardiac biomarkers and catecholamine level were not measured for this patient.

**Table 1:** Stress echocardiography details for case 1.

	**Exercise Stress ** **Echocardiogram**		**Dobutamine Stress ** **Echocardiogram**	
	
	**Rest**	**Peak Stress**	**Rest**	**Peak Stress**
**HR**	98	168	96	162
**BP**	128/68	222/110	118/66	138/72
**ECG**	NSR	<1 mm Jelevation in II, III, aVF	NSR	Sinus Tachycardia
**LVEF**	60%	50%	60%	70%
**WMSI**	1.00	2.00	1.00	1.00
**WMA**	None	Apical akinesis and mid-ventricular hypokinesis	None	None

(LVEF = Left ventricular ejection fraction, WMSI = Wall motion score index, NSR = Normal sinus rhythm)

Real time myocardial contrast echocardiography was performed along with DSE during an established research protocol to assess myocardial perfusion using continuous infusion of Definity^® ^(BMS, USA) at a rate of 200 ml/hour. Micro-bubble destruction replenishment imaging sequences were performed using iE33 echo system (power modulation – Philips Medical Systems). Qualitative perfusion analysis demonstrated normal myocardial blood flow reserve in all 16 segments at rest and peak stress, indicating absence of any critical coronary artery lesion.

## Case 2

**Figure 2:**
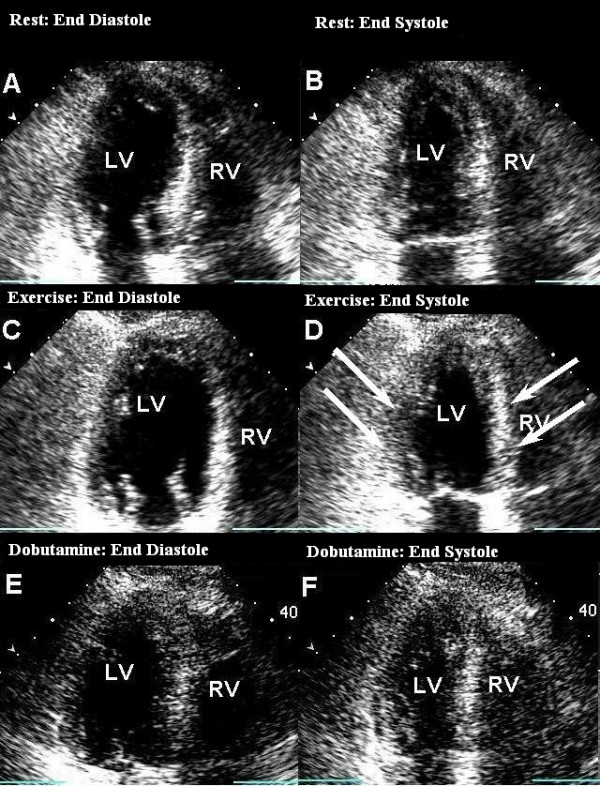
**Treadmill Exercise test showing basal akinesis, and mid-LV hypokinesis, with normal apical contractility during peak stress**. Images A and B were taken during rest, and C and D are at peak stress, A and C represent diastolic frames, B and D represent systolic frames. Image D shows basal and mid-LV wall motion abnormality. Images E (end-diastole) and F(end-systole) are from Dobutamine stress test at peak stress. Image F is an end systolic frame showing good contraction of all the segments as compared to D. (LV: Left Ventricle, RV: Right ventricle).

A 75 year old man with cardiac risk factor of hyperlipidemia, on simvastatin 10 mg daily, underwent treadmill echocardiographic testing as a pre-operative evaluation for right internal carotid artery stenting. Patient achieved 4.1 METS during the standard Bruce protocol, and denied any chest discomfort. Test was terminated due to fatigue and leg distress. New regional WMA were noted in mid and basal segments, with basal segments being akinetic (figure 2), while apical segments had normal wall motion. Please refer to table 2 for the detailed result of stress echocardiograms. The abnormal WMA was suspected to be due to a hypertensive response, and a repeat DSE was performed with standard protocol on the next day. This study failed to reveal any WMA. Cardiac biomarkers and catecholamine level were not measured for this patient.

**Table 2:** Stress echocardiography details for case 2.

	**Exercise Stress ** **Echocardiogram**		**Dobutamine Stress ** **Echocardiogram**	
	
	**Rest**	**Peak Stress**	**Rest**	**Peak Stress**
**HR**	85	121	83	123
**BP**	102/66	184/84	108/66	120/74
**ECG**	NSR	<1 mm depression in V_4_	NSR	Sinus Tachycardia
**LVEF**	60%	55%	60%	70%
**WMSI**	1.00	1.63	1.00	1.00
**WMA**	None	Basal akinesis	None	None

(LVEF = Left ventricular ejection fraction, WMSI = Wall motion score index, NSR = Normal sinus rhythm)

## Discussion

Both of these cases suggest that actual, or relative, transient hypertensive response during 'exercise' stress may give rise to altered myocardial contractility resulting in false positive results. Exercise is a well known situation resulting in catecholamine release which can subsequently lead to subendocardial microvascular ischemia [2]. A marked increase in afterload during exercise due to hypertensive response could precipitate WMA. Another possible mechanism could be transient coronary vasospasm due to hyperventilation during exercise stress test [6]. But again, this cannot explain specific apical and basal segment akinesis. In our cases, hypothesis of hypertension induced transient WMA is supported by the temporal association of the events, and the absence of WMA on subsequent pharmacologic echocardiography testing when BP was not elevated. There was no structural heart disease evident on baseline echocardiogram in both patients.

Studies and reports have shown that hypertension can cause dynamic left ventricular outflow tract obstruction evoked by exercise leading abnormal wall motion [7,8]. Hypertension induced left ventricular hypertrophy causes alterations in the sub-endocardial microvasculature due to medial hypertrophy and peri-arteriolar fibrosis [9]. These changes, in turn, leads to WMA in the absence of coronary macro-angiopathy. Dobutamine is a direct acting inotropic agent whose primary activity results for direct stimulation of cardiac β receptors with minimal hypertension. Systemic vascular resistance is usually reduced with administration of dobutamine [10].

Previous studies have suggested that hypertensive response during exercise stress testing can cause false positive WMA in more than one coronary arterial territory [1]. Our patients fit into the description of transient stress cardiomyopathy, leading to WMA in different regions of LV. It cannot be labeled as TC due to lack of evidence of absence of CAD on coronary angiography. The first case represents TC like contractile pattern. Second case represents 'inverted' TC like pattern. Limitations to our observations include: 1) Coronary angiography was not performed in both the patients due to low suspicion for CAD, and lack of clinically justifiable indication, after observing the normal DSE response 2) Plasma catecholamine levels were not measured.

There is only one case report published describing stress response to exercise leading to TC [11]. This report lacks hemodynamic details during stress testing. To our knowledge, our report is the first to describe development of Takotsubo-like contractile pattern secondary to hypertensive response during exercise stress testing. It will be unfair to label it with 'stress induced cardiomyopathy' as this usually lasts for days. We strongly suspect that this kind of response is more prevalent, but under-reported, and/or misinterpreted. At present, the reduced specificity of WMA development during hypertensive stress response presents a clinical dilemma. Repeat pharmacologic stress test before proceeding with cardiac catheterization may be considered in evaluating such cases. Simultaneous myocardial function *and perfusion *imaging during contrast stress echocardiography may have a potential role in differentiating a false positive WMA due to hypertensive response, from true positive WMA due to epicardial CAD.

## Competing interests

The authors declare that they have no competing interests.

## Authors' contributions

AD and SSA has made substantial contributions to conception, editing images, and acquisition of data, literature search, participated in the sequence alignment of content and drafted the manuscript. MB and JKO were involved in echocardiography interpretations. SLM was involved in literature search, echocardiography results interpretations, drafting the manuscript and revising it critically for important intellectual content. All authors read and approved the final manuscript.
